# Establish new formulas for the calculation of renal and isthmus depth in horseshoe kidney

**DOI:** 10.1097/MD.0000000000014916

**Published:** 2019-03-22

**Authors:** Guangyu Ma, Yingmao Chen, Mingzhe Shao, Jiahe Tian, Baixuan Xu

**Affiliations:** Department of Nuclear Medicine, Chinese PLA General Hospital, Beijing, China.

**Keywords:** glomerular filtration rate (GFR), horseshoe kidney, isthmus depth, radionuclide renography, renal depth

## Abstract

This study was performed to develop a new formula to estimate the renal and isthmus depth in horseshoe kidney, and to compare the new formula with previously published formulas.

Renal depth, isthmus depth, vertebral thickness, and total thickness (*T*, cm) of the body at the level of the kidneys were measured by CT in 124 adults. Their sex, age, height (*H*, cm), and weight (*W*, kg) were recorded. Multiple stepwise linear regression analysis was conducted. The 124 cases were divided into 2 random groups, of which the first group was used to derive a regressive formula and the second group was used to verify the formula and compare the formula with previously published formulas.

Multiple stepwise linear regression analysis showed that the important variables in estimating the depth of each kidney were the body weight (*W*, kg) and the total thickness (*T*, cm) of the body at the level of the kidneys. The important variables in estimating the depth of isthmus soft tissue and vertebral thickness were *W*, *T*, and age, *W*. The new formula was the following: right renal depth (cm) = 0.273 × *T* + 0.043 × *W* + 1.086 (*r* = 0.82, *P* < .05; standardized regressive coefficient: *T* = 0.500, *W* = 0.367), left renal depth (cm) = 0.245 × *T* + 0.041 × *W* + 0.676 (*r* = 0.83, *P* < .05; standardized regressive coefficient: *T* = 0.520, *W* = 0.353); isthmus depth (cm) = soft tissue depth + vertebral thickness, soft tissue depth (cm) = 0.144 × *T* + 0.044 × *W* + 0.536 (*r* = 0.58, *P* < .05; standardized regressive coefficient: *T* = 0.272, *W* = 0.335), vertebral thickness (cm) = 0.012 × age + 0.018 × *W* + 3.683 (*r* = 0.53, *P* < .05; standardized regressive coefficient: age = 0.326, *W* = 0.438). It is much better than the literatures.

The new renal depth estimation formula in horseshoe kidney that we derived by using multiple stepwise linear regression has greatly outperformed other 6 previously published formulas. Isthmus depth estimation formula can also get accurate results. Our new formula provides a more reliable and accurate renal and isthmus depth estimation and contributes to improving the methods used to estimate renal function from radionuclide renography in horseshoe kidney.

## 1 Introduction

Glomerular filtration rate (GFR) refers to the amount of ultrafiltrate kidneys generated per unit time, which is an important indicator of kidney function.^[1]^ Renal dynamic imaging with Tc-99m diethylenetriaminepentaacetic acid (DTPA) is an ideal method for the determination of GFR, also known as the Gates’ method.^[2]^ The accuracy of Gates’ method is affected by renal depth. Renal depth is often calculated by estimation formulas. Renal depth deviation can cause GFR error,^[3]^ a ±1 cm error in true kidney depth which may cause an 18% difference in GFR in adults.^[1]^

Most horseshoe kidney (HSK) patients have abnormal kidney rotation and fusion of the kidneys at the lower poles to form an isthmus, and its anatomical structure is different from the normal form.^[4]^ The existing 6 formulas^[1,5–9]^ are based on the normal form of the kidney. All the existing renal depth estimation formulas do not apply to HSK.^[4]^ At present, there is no formula for estimating renal depth in patients with HSK. In addition, there is no estimation formula for isthmus depth.

In this study, we developed a new formula to estimate the renal and isthmus depth in HSK, and then to compare the new formula with previously published formulas.

## 2 Materials and methods

### 2.1 Materials

**Figure 1:**
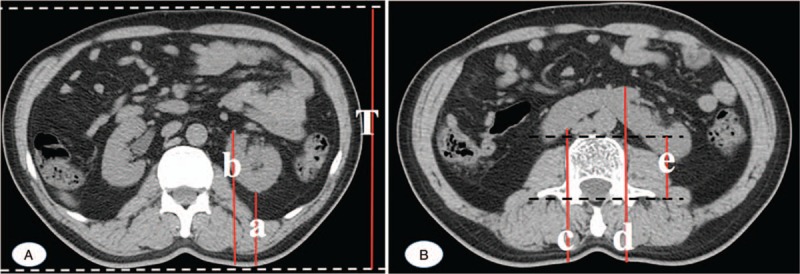
(A) CT scan showing skin to anterior and posterior renal surfaces at the level of the renal hilum. Renal depth was determined by averaging the anterior and posterior depths at the renal hilum: renal depth (cm) = (*a* + *b*)/2; *T* is total thickness of the body at the level of the kidneys. (B) Isthmus depth was determined by averaging the highest and lowest points on both sides of the isthmus vertebrae at the maximum cross sectional level of isthmus, isthmus depth (cm) = (*c* + *d*)/2; vertebral thickness (*e*) was determined from the anterior edge of the vertebral body to the transverse process of the vertebral body. Isthmus soft tissue depth = (*c* + *d*)/2 – *e*.

The study was approved by the Ethic Committee of Chinese PLA General Hospital and the written informed consent was obtained from each patient. The research objects of this article were patients undergoing routine clinical PET/CT or CT studies, and 124 HSK patents were selected. The patients were divided into 2 groups, of which the first group was used to derive a regressive formula and the second group was used to verify the formula. Patients with ascites, a single kidney, or masses that might distort the renal depth were excluded. Renal depth was determined by measuring from the skin on the posterior aspect of the renal at the renal hilum and then taking an average of these values to determine a mean depth (Fig. 1A).^[4]^ The total thickness (*T*, cm) of the body at the level of the kidneys was also measured by CT (Fig. 1A).^[4]^ The posterior part of the isthmus is composed of soft tissue and vertebral body. Because the attenuation coefficients of soft tissue and vertebral body to gamma rays are different, we need to obtain the depth of isthmus soft tissue and vertebral body thickness, respectively. Isthmus depth was determined by averaging the highest and lowest points on both sides of the isthmus vertebrae at the maximum cross-sectional level of isthmus (Fig. 1B). Vertebral thickness was determined from the anterior edge of the vertebral body to the transverse process of the vertebral body (Fig. 1B). The depth of the isthmus soft tissue is the difference between the isthmus depth and the vertebral body thickness. The following data were recorded: sex, age (year), height (*H*, cm), weight (*W*, kg), thickness (*T*, cm), renal depth, isthmus soft tissue depth, and vertebral thickness (Table 1).

**Table 1:**
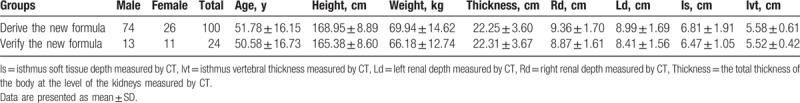
The general information of the data that was used to derive and verify the new formula.

### 2.2 Methods

A multiple linear stepwise regression analysis was carried out in 100 adult patients (ages from 19 to 92) to determine the relative importance of each of several variables to develop new regression formula for estimating renal depth. Variables under evaluation included sex, age, height, weight, weight/height, height/weight, thickness, thickness/weight, and weight/thickness. The new formula and the other 6 formulas were applied prospectively to a new set of 24 adult patients (ages from 21 to 80). The existing 6 formulas are as follows:

(Formula 1) Ma G.Y. formula^[1]^:
 
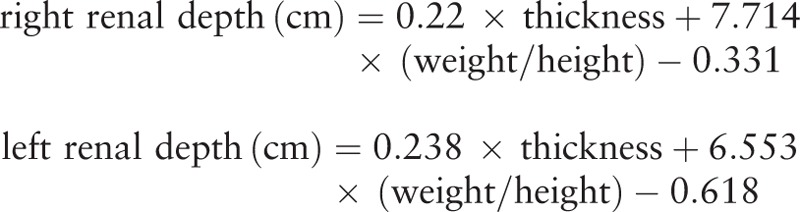


(Formula 2) Tonnesen formula^[5]^:
 
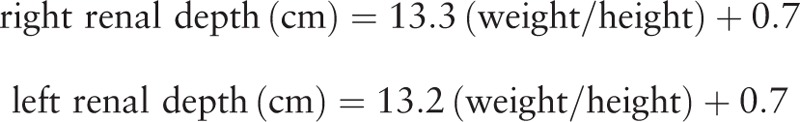


(Formula 3) Taylor formula^[6]^:
 
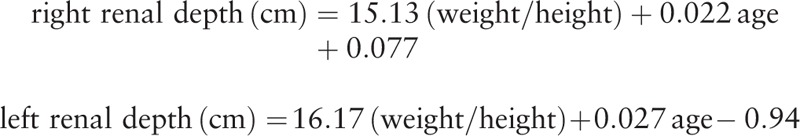


(Formula 4) Inoue formula^[7]^:
 
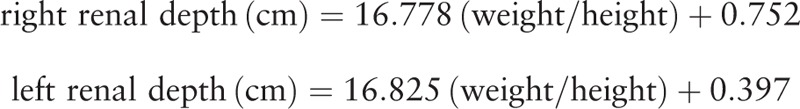


(Formula 5) Li Q. formula^[8]^:
 
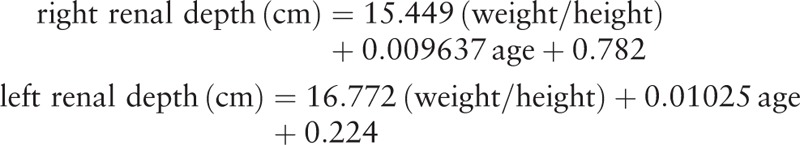


(Formula 6) Xue J.J. formula^[9]^:
 
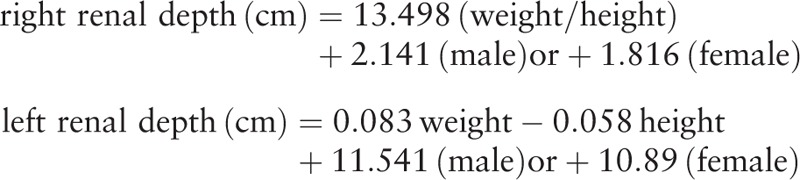


### 2.3 Statistical analysis

All data were expressed as the mean ± standard deviation of the mean (SD). A multiple linear stepwise regression analysis was carried out to obtain the regression equation. Correlation analysis was performed between estimated and CT measured renal depth and isthmus depth, and the correlation coefficient was calculated. In addition, the mean difference between the estimated and CT measured renal depth and isthmus depth was compared.

## 3 Results

### 3.1 New formula

Multiple stepwise linear regression analysis showed that the important variable in estimating the depth of each kidney was body weight and the total thickness of the body at the level of the kidneys. The important variables in estimating the depth of isthmus soft tissue and vertebral thickness were *W*, *T*, and age, *W*. The new formula was as follows: right renal depth (cm) = 0.273 × *T* + 0.043 × *W* + 1.086 (*r* = 0.82, *P* < .05; standardized regressive coefficient: *T* = 0.500, *W* = 0.367), left renal depth (cm) = 0.245 × *T* + 0.041 × *W* + 0.676 (*r* = 0.83, *P* < .05; standardized regressive coefficient: *T* = 0.520, *W* = 0.353); isthmus depth (cm) = soft tissue depth + vertebral thickness, soft tissue depth (cm) = 0.144 × *T* + 0.044 × *W* + 0.536 (*r* = 0.58, *P* < .05; standardized regressive coefficient: *T* = 0.272, *W* = 0.335), vertebral thickness (cm) = 0.012 × age + 0.018 × *W* + 3.683 (*r* = 0.53, *P* < .05; standardized regressive coefficient: age = 0.326, *W* = 0.438), where *W* is the body weight (kg) and *T* is the total thickness (cm) of the body at the level of the kidneys.

### 3.2 Correlation analysis

**Figure 2:**
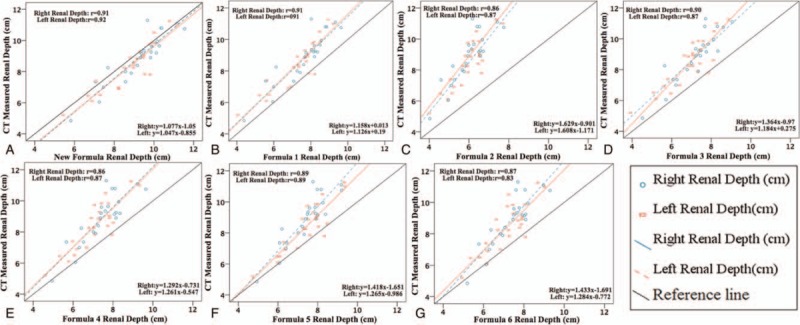
Relationship between estimated and measured renal depth in validation data.

**Figure 3:**
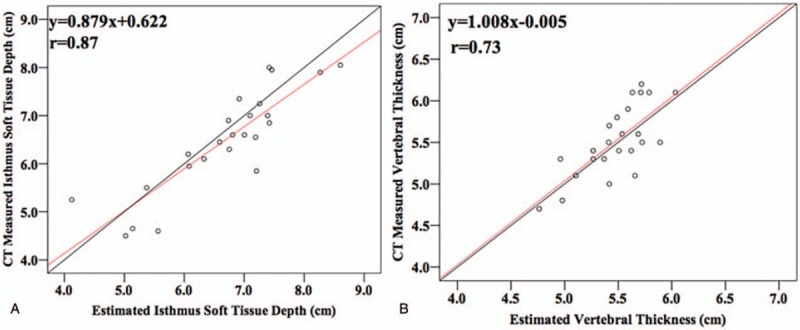
Relationship between estimated and measured isthmus soft tissue depth and vertebral thickness in validation data.

There was a strong and significant correlation between estimated and actual renal depth in the validation data. But the new formula is better than the other 6 formulas; the correlation coefficients were 0.91 for right renal and 0.92 for left kidney (Fig. 2A). The estimated left renal depth from formula 6 and actual renal depth are poorly correlated, and the correlation coefficients were 0.87 for right renal and 0.83 for the left kidney (Fig. 2G). The estimated isthmus soft tissue depth and vertebral thickness had good correlation with measured data; the correlation coefficients were 0.87 for isthmus soft tissue depth and 0.73 for vertebral thickness (Fig. 3).

### 3.3 Renal depth comparison

The results of the renal depth measurement on the CT are presented in Table 1. The prediction of renal depth using the new formula was successful, and the mean predicted of the depth was close to the mean measured depth, about 0.39 cm (Table 2). The performance of the new formula was much better than the other 6 formulas.

**Table 2:**
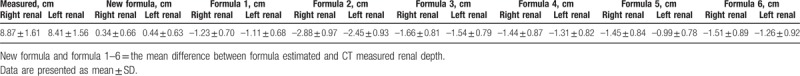
CT measured renal depth and the mean difference between estimated and actual renal depth in the validation data.

From Table 2 we can find that formulas 1 to 6 tended to underestimate renal depth for both kidneys, this result is consistent with a previous study.^[4]^ Formula 2 results are significantly lower than CT measured renal depth, underestimated about −2.67 cm. Although there was a strong and significant correlation between the estimated renal depth from formulas 1 and 3 to 6 and CT measured renal depth, the mean difference deviation is more than −1.22 cm which was unacceptable.

### 3.4 Isthmus depth comparison

The results of the isthmus soft tissue depth and isthmus vertebral thickness measurement on the CT are presented in Table 1. Both isthmus soft tissue depth and vertebral thickness estimated formulas can accurately estimate the depth and thickness. The mean difference between estimated and actual date was 0.19 ± 0.53 cm and −0.04 ± 0.29 cm, respectively (Table 3).

**Table 3:**
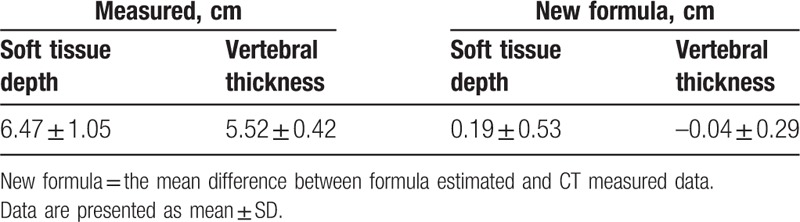
CT measured isthmus soft tissue depth and vertebral thickness and the mean difference between estimated and actual date in the validation data.

## 4 Discussion

HSK is a congenital abnormality in 1 of every 400 to 1000 individuals, and the incidence in men is twice compared with that in women.^[10]^ HSK patients always present with genitourinary and extragenitourinary congenital abnormalities, such as vascular abnormalities.^[11]^ They are prone to a variety of complications, such as stone disease, ureteropelvic junction (UPJ) obstruction, trauma, infection, and a variety of benign and malignant tumors.^[12–15]^ For HSK patients and patients with kidney diseases, it is important to accurately evaluate renal function to determine a suitable treatment plan.^[16]^ Accurate assessment of GFR is essential for interpreting symptoms and signs and for drug dosing, detecting and managing kidney disease and assessing prognosis.^[17]^

Gates’ method is often used for determination of GFR. The renal depth is important in determining the attenuation coefficient used to calculate kidney function from scintigraphic scans.^[4]^ Estimation formula is commonly used to calculate the renal depth in clinical work. The previous study found that GFR measured by ^99m^Tc-DTPA renal dynamic imaging is significantly lower than estimated GFR which was estimated by the Chronic Kidney Disease Epidemiology Collaboration (CKD-EPI) equation in HSK patients.^[16]^ The first reason is that the existing estimation formulas cannot accurately estimate the renal depth. The results showed that the existing estimation formulas significantly underestimated the renal depth in HSK, at least −1.22 cm. Second, there is no estimation formula for isthmus depth. Third, the linear attenuation coefficient of the isthmus is different from that of the kidney. Because the posterior part of the isthmus is composed of soft tissue and vertebral body, the attenuation of the vertebral body to the gamma ray was significantly higher than that of the soft tissue. Fourth, the method of delineation of region of interest in patients with horseshoe kidney should be different from normal shape.

In this study, we first established a new formula based on the patients with HSK to estimate the renal and isthmus depth in patients with HSK. In renal stepwise regression equations derived process, *T* was the first one to be introduced into the regression equation and *W* was the second one. *H* and *W*/*H* had no contribution to the regression equation. It is different from the existing renal depth estimation formulas. In isthmus soft tissue depth stepwise regression equations derived process, *W* was the first one to be introduced into the regression equation and *T* was the second one. In vertebral thickness stepwise regression equations derived process, *W* was the first one to be introduced into the regression equation and age was the second one. The results showed that the new formula performs well in the correlation coefficients and the mean difference deviation. It can accurately assess the renal and isthmus depth in patients with HSK, and it can be used in clinical.

## 5 Conclusion

The formulas in the literatures are based on the normal form of the kidney, and they do not apply to HSK. We obtained the new formula based on the patients with horseshoe kidney. Incorporation of the new formula into camera-based protocols to determine renal clearances can acquire more accurate measurements of renal function. Our next work is to develop a region of interest mapping method suitable for horseshoe kidney patients and obtain the isthmus attenuation coefficient.

## Acknowledgment

We thank Yang Qiao and Chen Jia Sheng for their valuable support.

## Author contributions

**Conceptualization:** Jiahe Tian, Baixuan Xu.

**Data curation:** Guangyu MA.

**Formal analysis:** Guangyu MA, Yingmao Chen.

**Investigation:** Guangyu MA.

**Methodology:** Guangyu MA, Yingmao Chen, Baixuan Xu.

**Project administration:** Guangyu MA, Baixuan Xu.

**Resources:** Guangyu MA, Mingzhe Shao.

**Software:** Mingzhe Shao.

**Supervision:** Baixuan Xu.

**Validation:** Baixuan Xu.

**Visualization:** Baixuan Xu.

**Writing – original draft:** Guangyu MA.

**Writing – review and editing:** Guangyu MA.
